# UK Adults’ Exercise Locations, Use of Digital Programs, and Associations with Physical Activity During the COVID-19 Pandemic: Longitudinal Analysis of Data From the Health Behaviours During the COVID-19 Pandemic Study

**DOI:** 10.2196/35021

**Published:** 2022-06-21

**Authors:** Verena Schneider, Dimitra Kale, Aleksandra Herbec, Emma Beard, Abigail Fisher, Lion Shahab

**Affiliations:** 1 Department of Behavioural Science and Health University College London London United Kingdom; 2 SPECTRUM Research Consortium Edinburgh United Kingdom; 3 Clinical, Educational and Health Psychology University College London London United Kingdom

**Keywords:** pandemic, physical activity, longitudinal, United Kingdom, digital health, tele-exercise, moderate-to-vigorous physical activity, muscle-strengthening activity, COVID-19, home-based exercise, exercise, telemedicine, longitudinal, health behavior, behavior, data

## Abstract

**Background:**

Digital physical activity (PA) program use has been associated with higher PA guideline adherence during COVID-19 pandemic confinements. However, little is known longitudinally about exercise locations (inside vs outside the home environment), digital program use, and their associations with moderate-to-vigorous PA (MVPA) and muscle-strengthening activities (MSAs) during the pandemic.

**Objective:**

The aims of this study were to assess the relationship between exercise location and use of digital programs with PA guideline adherence during the COVID-19 pandemic, describe how individuals exercised inside and outside of their home environments, and explore which sociodemographic and contextual factors were associated with exercise locations and digital PA program use.

**Methods:**

Active UK adults (N=1938) who participated in the 1-month follow-up survey of the Health Behaviours During the COVID-19 Pandemic (HEBECO) study (FU1, June-July 2020) and at least one more follow-up survey (FU2, August-September; FU3, November-December 2020) reported exercise locations and types of exercises inside and outside their homes, including digital programs (online/app-based fitness classes/programs), MVPA, and MSA. Generalized linear mixed models were used to assess associations of exercise location and digital PA program use with PA guideline adherence (MVPA, MSA, full [combined] adherence), and predictors of exercise location and digital program use.

**Results:**

As the pandemic progressed, active UK adults were less likely to exercise inside or to use digital PA programs compared with periods of initial confinement: 61% (95% CI 58%-63%; weighted n=1024), 50% (95% CI 48%-53%; weighted n=786), and 49% (95% CI 46%-51%; weighted n=723) performed any exercise inside their homes at FU1, FU2, and FU3, respectively. At FU1, FU2, and FU3, 22% (95% CI 21%-25%; weighted n=385), 17% (95% CI 15%-19%; weighted n=265), and 16% (95% CI 14%-18%; weighted n=241) used digital PA programs, respectively. Most participants who exercised inside already owned indoor equipment, used digital PA programs, or had their own workout routines, whereas MVPA and gentle walking were the most common exercise types performed outside the home. Being female, nonwhite, having a condition limiting PA, indoor exercising space, a lower BMI, and living in total isolation were associated with increased odds of exercising inside the home or garden compared with outside exercise only. Digital PA program users were more likely to be younger, female, highly educated, have indoor space to exercise, and a lower BMI. While exercising inside was positively associated with MSA and exercising outside was positively associated with MVPA guideline adherence, both inside (vs outside only) and outside (vs inside only) activities contributed to full PA guideline adherence (odds ratio [OR] 5.05, 95% CI 3.17-8.03 and OR 1.89, 95% CI 1.10-3.23, respectively). Digital PA program use was associated with a higher odds of MSA (OR 3.97-8.71) and full PA (OR 2.24-3.95), but not with MVPA guideline adherence.

**Conclusions:**

During the COVID-19 pandemic, full PA guideline adherence was associated with exercising inside and outside of one’s home environment and using digital PA programs. More research is needed to understand the reach, long-term adherence, and differences between digital PA solutions.

## Introduction

Insufficient physical activity (PA) and sedentary behavior are among the leading risk factors for premature mortality and chronic conditions, and present a global public health concern [1,2]. The World Health Organization (WHO) recommends 150 weekly minutes of moderate-to-vigorous PA (MVPA) and two weekly sessions of muscle-strengthening activities (MSAs) for adults [2]. However, approximately one-third of the English population aged 16 years or over did not meet MVPA guidelines in 2019 [3], and the prevalence of MSA guideline adherence is reported to be as low as 10%-30% across countries [4]. The economic costs of insufficient PA for the National Health Service England are estimated at £450 million (~US $568 million) a year [5]. The COVID-19 pandemic confinements have been linked to worldwide declines in PA levels [6] and changes in individuals’ exercise habits [7]. To mitigate the impact of the COVID-19 pandemic and to inform responses to future pandemics, it is important to understand what helps individuals to exercise sufficiently during restrictions and at different phases of the pandemic, and whether digital PA programs can support this activity.

Population-level negative impacts of the initial pandemic confinements on PA and sedentary behavior have been reported in multiple observational studies [6,8-10]. In England, the proportion of active individuals aged 16 years or older dropped by 7.1% to 58.2% during the first UK lockdown [11]. However, despite the frequently reported population-level decline, the impact was not equal across demographic groups, as some individuals were able to maintain or even increase PA levels [12]. The groups most strongly impacted by PA declines were individuals with higher baseline PA levels [10,13,14], and those who were employed [12], in lower socioeconomic positions [3,11,12,15-17], female [16-18], nonwhite [3,11,19], living alone [17], with higher BMI [17], or living with a health condition [3,17,20]. Although some studies reported negative impacts for older age groups [16,18], others found that younger adults were most strongly affected [3,10,12,21]. In addition, having access to space to exercise, at home or within the neighborhood [17,19], and living with others rather than alone [17] have been identified as protective factors against declines or low PA levels.

As exercise contexts changed due to the pandemic, PA habits have likely been disrupted [7]. Although this may pose a risk to healthy habits, new opportunities and contexts may also promote the uptake of beneficial behaviors. However, UK evidence on long-term PA changes throughout the pandemic is scarce and conflicting. Repeated cross-sectional data taking into account prepandemic seasonal activity trends suggest a partial recovery of the pandemic impact during the summer and autumn of 2020 compared to the first lockdown [11]. Longitudinal data suggest positive long-term changes in PA behaviors from lockdown into easing of restrictions [22] or continued PA declines into the autumn/winter 2020 [23].

While seasonal [24,25] and pandemic-specific barriers may affect outdoor PA and opportunities to exercise in gyms, leisure facilities, and organized sports, engagement in PA at home may be associated with fewer declines in PA. For example, exercising in one’s home or driveway was associated with higher MVPA in a cross-sectional study with US adults in the first 2 months of COVID-19 restrictions [19]. Different exercise locations lend themselves to different exercise types due to the availability of equipment and space. Thus, exercise locations may be differently related to MVPA and MSA. For example, exercises inside one’s home may have higher components of MSA due to strengthening exercises not requiring a lot of space. By contrast, exercises outside one’s home may have higher components of MVPA, as individuals are more likely to engage in aerobic activities such as brisk walking, running, or cycling. In the initial UK lockdown, half of those who exercised reported substantial changes in the form of exercise (none or some of the same exercises) [26]; however, little is known on how or if the forms of exercises changed over different phases of the pandemic and across seasons.

Additionally, the increasingly prevalent use of digital technologies such as web-based and smartphone-based programs and services, including apps, may provide additional support for the engagement in, and maintenance of, PA behavior. Studies conducted during the initial confinements suggest that users of digital support such as PA apps or online platforms were more likely to meet recommended PA guidelines [27,28] and less likely to experience a decrease in PA [16,29]. However, none of these studies used data from different phases of the pandemic beyond the initial confinements. Further, only one cross-sectional study conducted during the initial lockdown of the COVID-19 pandemic in Australia included measures of MSA [28]. This study reported that 39.5% of adults used some form of digital PA platform, of which streaming services (eg, YouTube, Instagram, and Facebook) and facilitated live or recorded online classes (eg, via Zoom) were the most frequently reported (42% and 31%, respectively). Compared with nonusers, digital PA platform users were 2.7-times more likely to meet WHO recommendations for both MVPA and MSA [28].

Although observational studies are limited regarding causal conclusions, describing naturally occurring behavior and associated factors in observational real-world studies can help hypothesis generation for further research and intervention development. By identifying factors associated with exercise locations and digital program use, different target groups, and potential barriers, facilitators, and risks relating to feasibility, reach, and adherence over time can be identified. Research conducted before and during the pandemic identified users of health apps or digital platforms as more likely to be female [28,30], younger [31,32], frequent smartphone users [30], with higher education and income [31], with a chronic condition [32], employed, and without home or caring duties [28]. However, little is known to date about factors associated with exercise locations and digital program use or about their associations with MVPA and MSA levels, and the changes in the ways of exercising across different phases of the pandemic.

To address this gap, the primary aim of this study was to assess the relationship between exercise location and use of digital PA programs with MVPA, MSA, and full guideline adherence among active UK adults during the COVID-19 pandemic (between May and December 2020). Secondary aims were to describe changes over the course of the COVID-19 pandemic in exercise location, how individuals exercised inside and outside of their homes, and to explore which sociodemographic and contextual predictors were associated with the choice of exercise locations and digital PA program use.

Thus, this study sought to answer the following research questions (RQs) using longitudinal data collected during the COVID-19 pandemic: (RQ1) What were the differences in exercise location and the ways of exercising in June-July, August-September, and November-December 2020 during the COVID-19 pandemic? (RQ2) What demographic and contextual factors were associated with exercise location and use of digital PA programs in June-July, August-September, and November-December 2020 in active UK adults during the COVID-19 pandemic? (RQ3) What was the association of exercise location and digital PA program use with PA guideline adherence (MVPA, MSA, and combined) in active UK adults in June-July, August-September, and November-December 2020 during the COVID-19 pandemic?

## Methods

### Design

This study analyzed longitudinal data from the Health Behaviours During the COVID-19 Pandemic (HEBECO) study [33]. Baseline data collection took place between April 23 and June 14, 2020, during the first UK-wide lockdown, with follow-up (FU) questionnaires sent out at 1 month (FU1; June-July 2020, lockdown/some lifts of restrictions), 3 months (FU2; August-September 2020, fewer restrictions), and 6 months (FU3; November-December 2020, country-specific lockdown/restrictions). Full details of the pandemic context for each data collection phase are described in Multimedia Appendix 1. As data on the key outcome variables were only collected as part of the core follow-up surveys, this study only included the three time points at FU1, FU2, and FU3. This study was preregistered on Open Science Framework [34] (see Multimedia Appendix 2 for changes to the protocol).

### Ethics Approval

Ethical approval was granted by University College London (UCL) Research Ethics Committee at UCL Division of Psychology and Language Sciences (CEHP/2020/579).

### Recruitment

Participants were recruited into the HEBECO survey through various channels such as paid advertisements, social media, charities, and partner organizations [33]. For the purposes of this study, non-UK residents or those who were completely physically inactive at all time points (MVPA=0 and MSA=0) were excluded. The latter criterion was a methodological consideration to reduce a risk of bias from including inactive participants in models of associations. The current survey was not set up to identify any previous download of digital programs but rather specifically asked about whether participants engaged in PA had used digital programs to do so. Since it is logically impossible to be simultaneously inactive while exercising using digital PA programs, including inactive participants would create a meaningless or inflated association between program use and PA guideline adherence. Thus, to ensure the results’ internal validity, eligible participants needed to have self-reported any MSA or MVPA for at least one time point.

### Measures

#### Outcome Measures

Full details and wording of measures can be found in the protocol [34] and Multimedia Appendix 3. All outcome variables were collected repeatedly during FU1, FU2, and FU3.

WHO PA guideline adherence (MVPA, MSA, and combined) was reported using validated questions based on the Behavioral Risk Factor Surveillance System 2015 [35,36]. MVPA was assessed by asking participants (1) how many times on average per week they had done a minimum of 15 minutes of MVPA (eg, brisk walking, jogging, dancing, cycling) and (2) how long (in minutes) an average session had been in the past month. Weekly average MVPA was defined as the product of these two variables. MSA was assessed by asking participants how many days per week on average they had done strength training in the past month. Three binary outcome variables were created to indicate individuals who met WHO MVPA recommendations (MVPA≥150 minutes/week) versus not, individuals who met WHO MSA recommendations (MSA≥2 sessions/week) versus not, and individuals who met both full recommendations versus not. The reported 2-week retest reliabilities of the MVPA and MSA measures are considered substantial (Cohen *κ*=0.67 and *κ*=0.85, respectively) and concurrent validities with activity logs are considered moderate (*κ*=0.41 and *κ*=0.52, respectively) [35].

Exercise location was assessed by asking participants who indicated engaging in any level of MVPA or MSA whether they had been exercising inside, outside, or both inside and outside their house/garden (items generated by the HEBECO study team). To account for the effects of doing any exercise either inside or outside one’s home environment with mutually exclusive categories, these were dichotomized into two variables: (1) any activity in the home environment versus only outside and (2) any activity outside of the home environment versus only inside.

Type of exercise was assessed by asking participants who indicated exercising inside or outside their home environments “What exercises are you usually doing inside/outside your house/garden?” Multiple answers were possible and were combined by creating the dichotomous variables (1) gentle walking (vs not), (2) MVPA activities such as any brisk walking/alternate walking-running/running/cycling/swimming (vs not), (3) any team/racket sports (vs not), (4) weightlifting (vs not), (5) online/app-based fitness classes/program (vs not), and (6) other (vs not) for activities outside the home environment. Activities inside the home environment were (1) exercise DVD (vs not), (2) online/app-based fitness classes/program (vs not), (3) using indoor exercise equipment that I already had (vs not), (4) using indoor exercise equipment that I bought/borrowed during COVID-19 (vs not), (5) doing bodyweight exercises without using an online class or app (ie, your own workout; vs not), and (6) other (vs not). Open-text responses on the “other” category were included in the FU1 and FU2 surveys only, precluding a systematic coding of these answers.

Use of digital PA programs was a dichotomous variable indicating individuals who had reported exercising using any “online/app-based fitness classes/program” (inside or outside) versus not.

#### Sociodemographic Predictors

Sociodemographic predictors collected at baseline were gender (female, others), age (<35 years as reference, 35-64 years, and ≥65 years), ethnicity (white, other), education (≥16 years, <16 years), health condition limiting PA (yes, no), and country of UK residence (England, other countries). Space to exercise comfortably inside one’s home or garden was only assessed at FU2 and FU3 and therefore dichotomized into no space on at least one time point (reference category vs all others).

#### Time-Variant Predictors

Repeatedly measured predictors were employment (full/part-time vs others), COVID-19–induced isolation (total vs some, general, no isolation [reference]), BMI (continuous), perceived risk of COVID-19 to one’s health (major-significant, lower), smoking (current, not), and alcohol consumption per week (>14, ≤14 units; [37]). Time was measured in months to account for the unequal time intervals between measurement points (1, 3, and 6 months) and centered at zero (0, 2, and 5). In addition to the key variables exercise location and digital PA program use, as described in the outcome measures, a time×exercise location interaction term was created to assess any differences in associations between location and PA guideline adherence over time.

### Statistical Analysis

Descriptive analyses and assumption checks were performed in SPSS 27.0. Weighted data were used to account for nonrandom sampling when describing the sample, and their exercise locations and types (RQ1). Weights account for population proportions of gender, age, ethnicity, household income, and country [38]. Descriptive statistics were calculated to describe the sample on key demographic and study variables. The analytic sample and participants lost to follow-up since baseline were compared on baseline characteristics using *t*-tests for continuous variables and *χ*^2^ tests for categorical variables. Descriptive statistics on exercise location, and activities inside and outside the home environment were computed as percentages per wave with cross-sectionally complete data.

RQ2 and RQ3 were assessed by running generalized linear mixed models (GLMMs) with dichotomous outcomes in R using the lme4 package [39]. First, linearity of the continuous predictors with the log of the outcome were checked by entering the predictor and its interaction effect with the log of itself into the model. According to Field [40], a significant interaction effect indicates a problem with linearity. As the continuous age variable was violating the linearity assumption, the categorical variable was used throughout, as specified in the Measurement section. Second, checks were run to ensure that multicollinearity was not present, which included inspection of the correlation matrix for correlations ≥0.8 and the calculation of variance inflation factors. Any variance inflation factor≥10 would have been considered problematic [40].

For RQ2, GLMMs were run to assess predictors of exercise location (exercising inside vs only outside and exercising outside vs only inside one’s home environment) and digital PA program use (binary logistic mixed model, reference: none). Repeated-measures (level-1) variables were nested within participants (level-2) and grand mean–centered. Univariate and fully adjusted models with random intercepts were run by including all the above-listed time-variant and -invariant predictors (except smoking and alcohol consumption). Similarly, GLMMs were run for RQ3, predicting MVPA, MSA, and full PA guideline adherence. First, unadjusted and adjusted models with time, the key predictors (exercise location, use of digital classes), and the interaction term with time were run. Second, models were fully adjusted for the remaining predictors. Sensitivity analyses were performed on a data set including only participants with complete data in all waves (FU1, FU2, and FU3). Significance thresholds in unadjusted models were Benjamini-Hochberg–adjusted to account for family-wise error [41].

In the absence of significant effects, Bayes factors (BFs) were computed using an online calculator [42] to distinguish insensitive data (1/3<|BF|≤3) from an absence of an effect (|BF|<1/3). Absolute BFs>3 were considered as moderate relative evidence for an effect. Based on associations of digital platform use with PA guideline adherence reported by Parker et al [28], half-normal distributions (eg, one-sided tests) with hypothesized effects of odds ratio (OR)=2.0 (MVPA), OR=3.3 (MSA), and OR=2.7 (full guidelines) were specified for RQ3.

## Results

### Sample Characteristics

Of the 2992 UK adults who participated in the baseline survey, 2363 (78.98%) started the FU1 survey. Of these, 14 (0.59%) moved out of the United Kingdom and 253 (10.71%) did not participate in any further follow-up. An additional 158 were excluded due to complete physical inactivity. Thus, the final analytic sample consisted of 1938 UK adults who provided a total of 5429 observations. When applying baseline weights to account for nonrandom sampling, the analytic sample was n=1680 (sample lost to follow-up n= 680).

Table 1 presents the baseline characteristics of the analytic sample and participants lost from baseline to FU1 (for unweighted estimates see Multimedia Appendix 4). The weighted analytic sample consisted of a higher proportion of individuals older than 64 years, of white ethnicity, and higher education, and a lower proportion of individuals living in total isolation, smokers, and individuals adhering to full PA guidelines at baseline. Further, the analytic sample had a significantly higher BMI than that of participants lost to follow-up.

**Table 1:** Baseline characteristics of the analytic sample and participants lost to follow-up (weighted population estimates, baseline weights).

Characteristic		Analytic sample (n=1680)	Sample lost to follow-up (n=680)	*P* value
**Age (years), weighted n (%)**				<.001
	<35	264 (15.7)	272 (40.0)	
	35-64	1087 (64.7)	349 (51.3)	
	>64	329 (19.6)	59 (8.7)	
Female, weighted n (%)		868 (51.7)	355 (52.2)	.81
White ethnicity, weighted n (%)		1529 (91.0)	558 (82.1)	<.001
16+ years of education, weighted n (%)		1146 (68.2)	432 (63.5)	.03
Employed, weighted n (%)		813 (48.4)	337 (49.6)	.62
Condition limiting PA^a^, weighted n (%)		240 (14.4)	107 (16.2)	.27
Living in England, weighted n (%)		1419 (84.5)	552 (81.2)	.05
Total isolation, weighted n (%)		93 (5.6)	66 (10.0)	<.001
High perceived risk from COVID-19, weighted n (%)		432 (25.9)	151 (22.8)	.11
Smoker, weighted n (%)		325 (19.4)	267 (39.3)	<.001
High alcohol consumption, weighted n (%)		288 (18.4)	127 (22.0)	.06
**Meeting WHO^b^** **PA recommendations at baseline, weighted n (%)**				
	MVPA^c^	676 (42.0)	248 (41.2)	.73
	MSA^d^	499 (31.0)	182 (30.1)	.70
	Both	265 (16.5)	121 (20.1)	.046
BMI, mean (SD)		26.5 (4.9)	25.6 (5.2)	<.001

^a^PA: physical activity.

^b^WHO: World Health Organization.

^c^MVPA: moderate-to-vigorous physical activity.

^d^MSA: muscle-strengthening activity.

### RQ1: Exercise Locations, Use of Digital PA Programs, and PA Behaviors

Table 2 presents descriptive data on exercise locations, use of digital PA programs, and WHO guideline adherence at the three time points (for unweighted estimates see Multimedia Appendix 5). Across time, most participants exercised only outside or both inside and outside their home environments (68%-74%), whereas fewer individuals exercised inside their home environments only (15%-18%). The proportions of individuals who did any exercise inside their home environments was 61% (95% CI 58%-63%), 50% (95% CI 48%-53%), and 49% (95% CI 46%-51%) at FU1 (June-July 2020), FU2 (August-September 2020), and FU3 (November-December 2020), respectively. While over one-fifth (23%, 95% CI 21%-25%) of active adults used digital PA programs at FU1, the proportions were 17% (95% CI 15%-19%) and 16% (95% CI 14%-18%) at FU2 and FU3, respectively. In addition, 18% of participants (95% CI 16%-20%) adhered to the full WHO guidelines at FU1, 13% (95% CI 12-15%) at FU2, and 13% (95% CI 12%-15%) at FU3.

**Figure 1:**
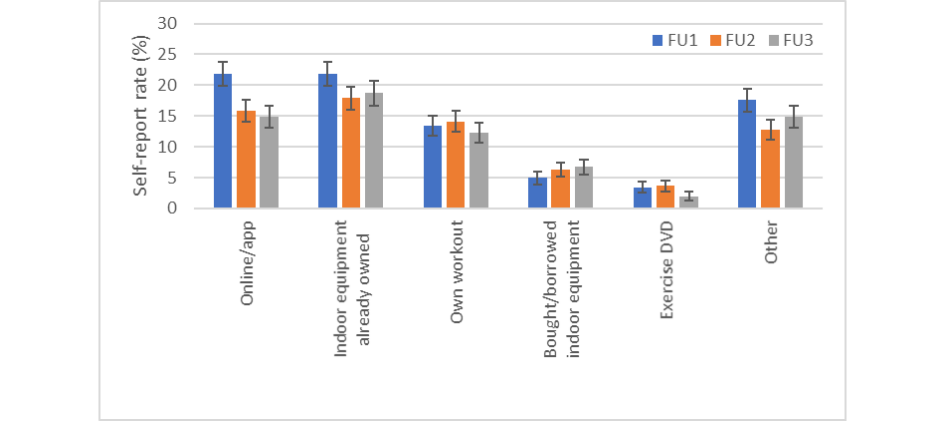
Exercise types inside one's home environment (weighted population estimates). FU: follow-up.

The most frequently reported ways of exercising inside one’s home environment were using already owned indoor equipment, digital PA programs, one’s own workout, or other (Figure 1). Other exercise types as indicated in open-text responses in FU1 and FU2 included gardening, do-it-yourself (DIY) activities, physiotherapy exercises, playing with children, Pilates, yoga, stretching, gymnastics, dancing, martial arts, and private personal trainer sessions. Less frequent were use of DVD or bought/borrowed equipment. The highest relative frequency of reporting the use of own indoor equipment and digital PA programs was observed at FU1 (June-July 2020).

**Figure 2:**
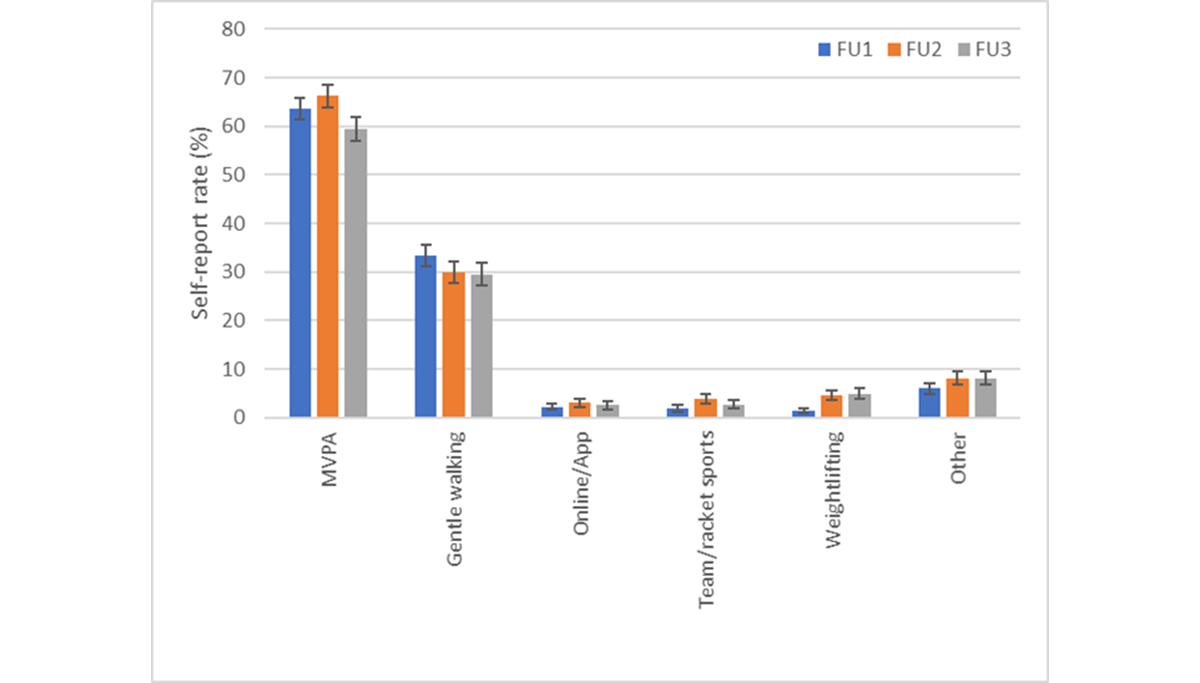
Exercise types outside one's home environment (weighted population estimates). FU: follow-up.

MVPA was the most frequently reported exercise type outside of the home environment, followed by gentle walking (Figure 2). Frequent open-text responses in the “other” category in FU1 and FU2 included horse riding or looking after horses, dog agility, water sports, climbing/hiking, golf, martial arts, the return to the gym, and various group and private trainer sessions. The highest relative frequency of MVPA was reported during FU2 (August-September 2020), a time of fewer restrictions, which also saw the highest relative frequency of team and racket sports compared with other time points.

**Table 2:** Exercise locations, use of digital programs, and meeting of World Health Organization (WHO) physical activity (PA) recommendations at follow-up 1 (FU1), follow-up 2 (FU2), and follow-up 3 (FU3).

Measure		FU1 (n=1725)^a^, weighted n (%)	FU2 (n=1587)^a^, weighted n (%)	FU3 (n=1535)^a^, weighted n (%)
**Exercise location^b^**				
	Inside home environment	312 (18.3)	238 (15.3)	238 (16.1)
	Outside home environment	526 (30.9)	614 (39.4)	522 (35.3)
	Both inside and outside	721 (41.9)	548 (35.1)	485 (32.8)
Use of digital PA programs^b^		385 (22.6)	265 (17.0)	241 (16.3)
**Meeting WHO recommendations^c^**				
	MVPA^d^	750 (44.0)	624 (39.9)	582 (39.3)
	MSA^e^	538 (31.6)	467 (29.9)	409 (27.7)
	Both	301 (17.7)	206 (13.2)	199 (13.5)

^a^Note that the n values differ from those in Table 1 due to different weights being applied (FU1, FU2, and FU3 weights vs baseline weights). Percentages are valid percentages (ie, excluding missingness).

^b^Missingness: FU1=24, FU2=27, FU3=56. Total N in exercise location includes active participants who dropped to inactivity at a certain time point (neither exercised inside nor outside the home environment); hence, percentages do not add up to 100%.^c^Missingness: FU1=22, FU2=24, FU3=56.

^d^MVPA: moderate-to-vigorous physical activity.

^e^MSA: muscle-strengthening activity.

### RQ2: Predictors of Exercise Locations and Digital PA Program Use

Factors associated with increased odds of exercising inside the home environment were female gender, having a condition limiting PA, having indoor space, and living in total isolation, whereas being in the 35-64–year age group (vs <35 years) and white ethnicity were associated with decreased odds of exercising inside (Table 3; for unadjusted analyses see Multimedia Appendix 6). A 1-point increase in BMI was associated with a 3% decrease in the odds of exercising inside versus outside the home environment only. However, the associations with age and BMI were not robust in the complete case analysis (Multimedia Appendix 7).

Associated factors with increased odds of exercising outside of the home environment were older age (>64 vs <35 years), higher education, having no condition limiting PA, not having indoor space to exercise, a lower perceived risk from COVID-19, and not living in total isolation. While the odds of exercising inside were significantly reduced at both FU2 and FU3 compared with FU1, time was not a significant predictor of the odds of exercising outside the home environment.

The odds of using digital PA programs decreased at FU2 and FU3 compared with FU1. Associated factors with increased odds of using digital PA programs were younger age, female gender, higher education, and indoor space. A 1-point increase in BMI was associated with an 8% decrease in the odds of using digital PA programs. Complete case analyses replicated these findings, except for education, which was nonsignificant (Multimedia Appendix 7).

**Table 3:** Fully adjusted generalized linear mixed model estimates of the predictors of exercising inside (vs only outside) or outside (vs only inside) the home environment, and of using digital physical activity (PA) programs (vs not) at follow-up 1 (FU1), follow-up 2 (FU2), and follow-up 3 (FU3).

Predictor		Exercising inside^a^			Exercising outside^a^		Digital PA program use^b^	
		OR^c^ (95% CI)		*P* value	OR (95% CI)	*P* value	OR (95% CI)	*P* value
**Time^d^ (reference: FU1)**								
	FU2		0.51 (0.42-0.62)	<.001	1.23 (0.89-1.70)	.20	0.47 (0.36-0.59)	<.001
	FU3		0.57 (0.47-0.70)	<.001	1.13 (0.81-1.58)	.46	0.50 (0.39-0.64)	<.001
**Age (years) (reference: <35 years)**								
	35-64		0.70 (0.49-1.00)	.048	1.60 (0.78-3.28)	.20	0.24 (0.14-0.40)	<.001
	>64		0.84 (0.54-1.31)	.45	3.44 (1.36-8.71)	.009	0.10 (0.05-0.21)	<.001
Female gender (reference: all other)			1.34 (1.03-1.75)	.03	1.17 (0.68-2.04)	.57	6.91 (4.46-10.71)	<.001
White ethnicity (reference: nonwhite)			0.46 (0.25-0.84)	.01	2.91 (0.89-9.48)	.08	0.51 (0.23-1.15)	.11
High education (reference: <16 years)			0.86 (0.58-1.27)	.45	2.89 (1.31-6.38)	.009	2.56 (1.37-4.76)	.003
Condition limiting PA (reference: none)			1.65 (1.12-2.45)	.01	0.31 (0.14-0.69)	.004	0.75 (0.41-1.34)	.33
England (reference: all other UK countries)			1.16 (0.82-1.65)	.41	0.67 (0.31-1.44)	.31	1.12 (0.65-1.92)	.68
Indoor space (reference: none)			6.12 (4.60-8.13)	<.001	0.56 (0.33-0.95)	.03	12.79 (8.30-19.69)	<.001
Employed (reference: not employed)			1.05 (0.82-1.34)	.72	1.52 (0.96-2.42)	.07	1.33 (0.94-1.87)	.11
BMI			0.97 (0.95-0.99)	.02	0.98 (0.93-1.03)	.37	0.92 (0.89-0.96)	<.001
High perceived risk of COVID-19 (reference: low)			1.30 (1.00-1.69)	.05	0.48 (0.30-0.77)	.002	0.95 (0.65-1.37)	.77
Total isolation (reference: not)			5.08 (2.18-11.82)	<.001	0.01 (0.00-0.03)	<.001	1.36 (0.63-2.95)	.43

^a^N=4492 observations, n=1772 individuals.

^b^N=4865 cases, n=1780 individuals.

^c^OR: odds ratio.

^d^Time violated the linearity assumption and was thus entered as a categorical variable.

### RQ3: Associations with PA Guideline Adherence

The odds of full guideline adherence decreased over time in active adults in the unadjusted analyses. However, the odds were attenuated when adjusting for the key predictors of location and digital program use (Multimedia Appendix 8), and were further attenuated to nonsignificance when adjusting for all remaining predictors in the analysis using the predictor exercising inside versus only outside the home environment (Table 4). Similarly, a significant decrease in the odds of adhering to MSA guidelines over time was attenuated when adjusting for the key predictors.

When fully adjusted for all other predictors, active adults exercising inside versus only outside their home environment had 5-times the odds of adhering to full PA guidelines (Table 4; for full tables with covariate estimates see Multimedia Appendix 9). Although they had 0.5-times reduced odds of adhering to MVPA guidelines, they had 9.7-times increased odds to adhere to MSA guidelines compared with adults who only exercised outside the home environment. The significant interaction between exercising inside and time indicated that the associations of exercising inside with MSA and full PA guideline adherence significantly differed over time, although this was not robust in complete case analyses (see Multimedia Appendix 10). Associations of exercising inside (vs outside the home environment only) with MSA and full PA guideline adherence were stronger at FU1 (OR 6.7, 95% CI 4.8-9.4 and OR 3.7, 95% CI 2.5-5.6, respectively) compared with FU2 (OR 4.5, 95% CI 3.3-6.0 and OR 2.9, 95% CI 2.0-4.1, respectively) and FU3 (OR 4.2, 95% CI 3.0-5.8 and OR 2.4, 95% CI 1.6-3.6, respectively; Multimedia Appendix 11).

Users of digital PA programs had 4-times the odds of adhering to MSA and 2.2-times the odds of adhering to full PA guidelines than active adults who did not use these programs. The association with MVPA was not significant. The BF of 0.37 indicated inconclusive evidence, although the OR<1 in the complete case analysis indicated an absence of an effect (BF=0.14; Multimedia Appendix 10).

When replacing the key variable exercising inside with exercising outside (vs inside the home environment only), exercising outside was associated with 4.4-times the odds of MVPA and 1.9-times the odds of full guideline adherence in active adults compared to those who only exercised inside their homes (Table 5; for unadjusted analyses, full tables with covariate estimates, and complete case analyses see Multimedia Appendices 8, 12, and 13 respectively). Further, people who exercised outside had 0.4-times the odds of adhering to MSA guidelines compared with those who exercised inside their home environment only. These associations did not significantly differ over time as indicated by the nonsignificant interaction. Users of digital PA programs were 8.7-times more likely to adhere to MSA and were 4-times more likely to adhere to full PA guidelines. Again, there was an absence of effect on MVPA guideline adherence (BF=0.14). Although time was associated with significantly decreased odds of meeting MVPA when entering exercising inside (vs outside only) as a predictor (Table 4), it was associated with decreased odds of meeting full guidelines in the model including exercising outside (vs inside the home environment only) as a predictor (Table 5).

**Table 4:** Generalized linear mixed model estimates predicting meeting moderate-to-vigorous activity (MVPA), muscle-strengthening activity (MSA), and full recommendations (vs not) at follow-up 1, 2, and 3; for key predictor exercising inside (vs outside the home environment only).

Predictor	MVPA^a^		MSA^a^		Full PA^b^ recommendations^a^	
	OR^c^ (95% CI)	*P* value	OR (95% CI)	*P* value	OR (95% CI)	*P* value
Time	0.81 (0.66-0.99)	.04	1.02 (0.80-1.30)	.86	0.84 (0.63-1.12)	.23
Exercising inside (reference: outside only)	0.54 (0.39-0.73)	<.001	9.70 (6.52-14.44)	<.001	5.05 (3.17-8.03)	<.001
Use of digital PA programs (reference: not)	1.10 (0.83-1.45)	.50	3.97 (2.92-5.38)	<.001	2.24 (1.60-3.13)	<.001
Time×location interaction	0.99 (0.91-1.08)	.85	0.87 (0.78-0.97)	.01	0.87 (0.76-0.99)	.04

^a^N=4439 observations, n=1769 individuals. Models fully controlled for age, gender, ethnicity, education, condition limiting PA, country, indoor space, employment, BMI, perceived risk of COVID-19, isolation status, smoking, and alcohol consumption. Bayes factor for nonsignificant associations with digital PA program use was 0.37 (MVPA).

^b^PA: physical activity.

^c^OR: odds ratio.

**Table 5:** Unadjusted and fully adjusted generalized linear mixed model estimates predicting meeting moderate-to-vigorous physical activity (MVPA), muscle-strengthening activity (MSA), and full recommendations (vs not) at follow-up 1, 2, and 3; for key predictor exercising outside (vs inside the home environment only).

Predictor	MVPA^a^		MSA^a^		Full PA^b^ recommendations^a^	
	OR^c^ (95% CI)	*P* value	OR (95% CI)	*P* value	OR (95% CI)	*P* value
Time	0.82 (0.67-1.01)	.06	0.84 (0.67-1.06)	.15	0.68 (0.52-0.89)	.005
Exercising outside (reference: inside only)	4.36 (2.87-6.63)	<.001	0.42 (0.27-0.65)	<.001	1.89 (1.10-3.23)	.02
Use of digital PA programs (reference: not)	0.95 (0.74-1.23)	.69	8.71 (6.39-11.86)	<.001	3.95 (2.84-5.50)	<.001
Time×location interaction	1.07 (0.94-1.23)	.30	1.10 (0.96-1.26)	.16	1.11 (0.93-1.32)	.26

^a^N=4439 observations, n=1769 individuals. Models fully controlled for age, gender, ethnicity, education, condition limiting PA, country, indoor space, employment, BMI, perceived risk of COVID-19, isolation status, smoking, and alcohol consumption. The Bayes factor for nonsignificant associations with digital PA program use was 0.14 (MVPA).

^b^PA: physical activity.

^c^OR: odds ratio.

## Discussion

### Principal Results

This study found strong associations between exercise location and digital program use with PA guideline adherence in a sample of active UK adults. Exercise location and digital program use showed different associations with MVPA, MSA, and full PA guideline adherence. Exercising inside the home environment was positively associated with MSA and exercising outside was positively associated with MVPA guideline adherence. Hence, exercising both inside and outside the home environment contributed to overall PA guideline adherence, while exercising only inside or only outside was associated with lower odds of adhering to full PA guidelines. Digital PA program use was also associated with MSA and full guideline adherence, but not with MVPA adherence. Furthermore, the results suggest that in the pandemic phases after the first initial confinements, active UK adults were less likely to exercise inside their home environment and to use digital PA programs. Users of digital PA programs were more likely to be younger, female, highly educated, have indoor space to exercise, and have a lower BMI. Most frequent exercise types inside the home environment included already owned indoor equipment, digital PA programs, one’s own workout, or other types (eg, gardening and DIY), whereas MVPA and walking were the most frequently reported exercise types outside the home environment.

These results are partially consistent with expectation and previous literature. In this study, most participants who exercised outside of their home environments engaged in MVPAs (such as running or cycling). As most MVPAs require space, MSAs may be more feasible for home-based exercise. Thus, it is not surprising that exercise location was differently associated with MVPA and MSA guideline adherence. However, previous cross-sectional research conducted during the first pandemic confinement in the United States found that exercising in one’s home, garage, yard, or driveway was associated with higher MVPA [19]. The finding that digital PA program use was positively associated with PA was consistent with previous literature [16,27-29]. Although the absence of an association with MVPA differed from the findings of Parker et al [28], they reported similar relative trends in the odds for MVPA (OR 2.0), MSA (OR 3.3), and full guideline adherence (OR 2.7) in a sample including inactive participants, with the strongest association found for MSA guideline adherence. In the current study, digital PA program use was one of the most frequently reported ways to exercise inside one’s home and likely has a stronger focus on MSA than MVPA due to feasibility in limited spaces. The attenuation of the ORs for both exercising inside the home environment and digital program use when controlling for each other in models predicting MSA and full guideline adherence further indicate a substantial amount of shared variance between these two predictors.

Regarding factors associated with PA digital program use in active adults, this study found that users of digital PA programs were more likely to be younger (<35 years), female, and highly educated, consistent with previous research [10,28,30,31]. Users were also more likely to have indoor space to exercise and to have a lower BMI. While digital PA programs may thus be able to target some groups at risk of insufficient PA during the pandemic (eg, women), they likely pose barriers for other disadvantaged groups who may not be able to benefit from digital solutions [43]. The pandemic has increased the already growing prepandemic health inequalities in the United Kingdom [44], and future research efforts should concentrate on how digital interventions can reach the groups most in need while addressing unintended adverse effects such as contextual, psychological, and socioeconomic access barriers [43]. Furthermore, while individuals with a condition limiting PA were more likely to exercise in their home environment, they were not more likely to use digital programs to exercise. This identifies a potential gap in targeted digital programs to address the needs of specific groups. Targeted digital interventions may be beneficial to individuals with conditions limiting PA and limited access to regular PA offers. In addition to identifying possible barriers, factors associated with digital program use may also represent differing preferences between groups. Further qualitative and quantitative research could examine the specific preferences, barriers, and facilitators associated with digital program use to help targeted intervention design.

Generally, fewer individuals adhere to MSA than to MVPA guidelines [45], and MSA has historically been neglected in guidelines and research [4]. Although this study found that active individuals who used digital PA programs and exercised inside their home environments were more likely to adhere to the MSA guidelines, the results also suggest that proportions dropped during the lifting of restrictions in the summer and reintroduction of restrictions in the autumn/winter of 2020. This drop also (partially) explained the decrease in MSA and full guideline adherence over time, which was seen in the attenuation of the effect of time when controlling for exercise location and digital program use. Health app engagement is often reported to be poorly sustained [46,47]. While the increased availability of digital PA programs and shift to home-based exercise may have presented an initial novelty, this may have become less attractive over the duration of the pandemic. It is also possible that the more strength-based activities at home were perceived as a substitute to aerobic activities rather than a complementary activity as advised in PA guidelines. Furthermore, exercising inside one’s home environment may be less motivating, for example, due to the lack of socialization. Future research should investigate the potential of home-based and digital exercise for promotion of MSA and full guideline adherence. It should also be explored whether different type of programs (eg, delivered live or on-demand) can foster different engagement and adherence rates.

### Limitations

This study is the first to investigate exercise locations, use of digital PA programs, and associations with PA guideline adherence, including MSA, during the COVID-19 pandemic in a longitudinal cohort of active UK adults. However, this study had some limitations.

First, all measures were self-reported. The agreement of self-reported with objectively measured PA varies substantially, and objective measures are often believed to be more accurate [48]. However, objective measures such as wearables are designed to track MVPA [49] and are hence less suitable to track home-based and strengthening activities [4]. Thus, future research should use both subjective and objective measures to account for a missing gold standard to capture both MVPA and MSA. Further, by using longitudinal data, any systematic measurement biases are corrected for as they would be expected to apply across waves. A second limitation of this study is its limited external validity due to the nonrepresentative sample, which was likely aggravated by attrition from baseline to FU3. Further, while the inclusion of inactive participants was considered methodologically problematic, their exclusion limited the generalizability of the results and may explain differences to the findings of other studies [28]. Considering the survey questions and aims in this study, and the bias of analyzing the full sample (as described in the Methods section), the adopted approach was deemed the most appropriate to answer this study’s research questions. However, the present results can therefore not contribute to hypotheses about facilitators to exercise in inactive adults. Future research may seek to assess the association of availability (eg, download) of digital programs or apps with PA guideline adherence including inactive participants. Third, the data are observational and thus preclude causal conclusions. The association between exercise locations and digital program use with PA outcomes have plausible alternative explanations through third variables such as overall PA motivation, self-efficacy, and prepandemic PA levels, which were not assessed in this study. Equally, this study was not set up to investigate potential mediating mechanisms to explain this link. Future experimental intervention research should investigate causal links between digital PA program use and PA, and assess mediating mechanisms. Finally, the measurement of digital PA programs (online or app-based fitness classes/programs) did not provide a clear definition of different type of programs (such as recorded/on-demand or live video–based programs) and did not explicitly include other forms of digital PA such as digitally conducted personal training sessions. Further research would benefit from distinguishing different types of digital solutions and investigating how they can implement different behavior change techniques [50].

### Conclusions

This study found that exercising both inside and outside the home environment and the use of digital programs to exercise were associated with full WHO PA guideline adherence in active adults during the pandemic. Digital PA programs may be suitable to support home-based MSA and thus support full guideline adherence. However, usage prevalence dropped during the first 6 months of pandemic restrictions. It is recommended that future research should further investigate the role of different digital PA interventions to promote PA and program adherence, using experimental designs and representative samples.

## Appendix

Multimedia Appendix 1 Pandemic context: UK restrictions on exercising in gyms and organized sports at Health Behaviours During the COVID-19 Pandemic (HEBECO) data collection waves.

Multimedia Appendix 2 Changes to the protocol.

Multimedia Appendix 3 Details of measures.

Multimedia Appendix 4 Unweighted baseline characteristics of the analytic sample and participants lost to follow-up.

Multimedia Appendix 5 Unweighted descriptive statistics: exercise locations, use of digital programs, and meeting of WHO PA recommendations at FU1, FU2, and FU3.

Multimedia Appendix 6 Unadjusted estimates: predictors of exercising inside (vs only outside), outside (vs only inside) the home environment, and of using digital PA programs (vs not) at FU1, FU2, and FU3.

Multimedia Appendix 7 Complete case analysis: predictors of exercising inside (vs only outside), outside (vs only inside) the home environment, and of using digital PA programs (vs not) at FU1, FU2, and FU3.

Multimedia Appendix 8 Predictors of meeting MVPA, MSA, and full recommendations (vs not) at FU1, FU2, and FU3. GLMM estimates with key predictors for unadjusted models, adjusted using exercising inside (vs outside the home environment only, Model 1) and using exercising outside (vs inside the home environment only, Model 2).

Multimedia Appendix 9 Predictors of meeting MVPA, MSA, and full recommendations (vs not) at FU1, FU2, and FU3. Fully adjusted GLMM estimates with key predictor exercising inside (vs outside the home environment only).

Multimedia Appendix 10 Complete case analysis: predictors of meeting MVPA, MSA, and full recommendations (vs not) at FU1, FU2, and FU3. GLMM estimates with key predictors only and using exercising inside (vs outside the home environment only).

Multimedia Appendix 11 Posthoc analysis: associations of exercising inside the home environment with PA guideline adherence at FU1-FU3; results from fully adjusted binary logistic regression models.

Multimedia Appendix 12 Predictors of meeting MVPA, MSA, and full recommendations (vs not) at FU1, FU2, and FU3. Unadjusted and fully adjusted GLMM estimates with key predictor exercising outside (vs inside the home environment only).

Multimedia Appendix 13 Complete case analysis, predictors of meeting MVPA, MSA, and full recommendations (vs not) at FU1, FU2, and FU3. GLMM estimates with key predictors only and using exercising outside (vs inside the home environment only).

## Abbreviations

BF: Bayes Factor
DIY: do it yourself
FU1: 1-month follow-up survey
FU2: 3-month follow-up survey
FU3: 6-month follow-up survey
GLMM: generalized linear mixed model
HEBECO: Health Behaviors During the COVID-19 Pandemic study
MSA: muscle-strengthening activities
MVPA: moderate-to-vigorous physical activity
OR: odds ratio
PA: physical activity
RQ: research question
UCL: University College London
WHO: World Health Organization
