# Supplementary material for: UK Adults’ Exercise Locations, Use of Digital Programs, and Associations with Physical Activity During the COVID-19 Pandemic: Longitudinal Analysis of Data From the Health Behaviours During the COVID-19 Pandemic Study
Source: JMIR Form Res. 2022 Jun 21;6(6):e35021. doi: 10.2196/35021 (PMC9217149; doi:10.2196/35021)
Supplement: Multimedia Appendix 1 [file formative_v6i6e35021_app1.docx]

## Multimedia Appendix 1 – Pandemic context: UK restrictions on exercising in gyms and organized sports at HEBECO data collection waves

|  | **England** | **Scotland** | **Wales** | **Northern Ireland** |
| --- | --- | --- | --- | --- |
| **Baseline (23 April – 14 June 2020)** UK-wide lockdown (partial easing on 13 May and 1 June) | - Beginning of lockdown: Exercise as one of few exceptions for leaving one’s home. - From 13 May: Driving to public spaces and spending time outdoors permitted. - 1 June: Outdoor gathering of up to six people, including exercising outdoors with up to five others allowed. - Closures of all retail and leisure businesses throughout (including gyms). | | | |
| **FU1 (05 June – 26 July 2020)** Easing of lockdown | - Outdoor gyms and outdoor pools permitted to reopen; indoor gyms remained closed in England, Scotland and Wales. - In Northern Ireland only, indoor gyms were permitted to reopen from 10 July. | | | |
| **FU2 (06 August – 29 September)** Few restrictions | - Indoor gyms, swimming pools and sports facilities permitted to reopen in England, Wales and Northern Ireland (including classes with reduced class sizes and organized team sports). - Gyms and pools reopened in Scotland from September 2020. | | | |
| **FU3 (04 November – 29 December 2020)** Country-wide lockdowns and local tier systems (majority of the countries in the higher tiers) | 2^nd^ lockdown (5 November – 2 December): Outdoor exercise allowed; closure of leisure facilities (including gyms).  Tier system from 2 December: Indoor classes prohibited in tier 3; introduction of a stricter tier 4 with closures of gym from 20 December. | 5-level tier approach (from 2 November): Outdoor sports allowed; indoor exercise classes prohibited from level 3; gyms/indoor leisure facilities closed at level 4. | Firebreak lockdown (23 October – 9 November): Closure of leisure businesses (including gyms).  From 9 November: Reopening of gyms, organized indoor and outdoor activities permitted. | Circuit breaker lockdown (16 October - 27 November): Gyms remained open; indoor classes or organized contact sport prohibited.  Renewed restrictions from 27 November: Closure of all gyms/leisure facilities.  11 December: Reopening of gyms/indoor leisure including indoor and outdoor classes. |

Sources

1. BBC. Coronavirus: Quarantine changes come into effect in NI. 2020, July 10; Available from: https://www.bbc.com/news/uk-northern-ireland-53354520.
2. BBC. Coronavirus: Scottish gyms and pools to reopen. 2020, August 31; Available from: https://www.bbc.com/news/uk-scotland-53964856.
3. BBC. Covid: Wales to go into ‘firebreak’ lockdown from Friday. 2020, October 19; Available from: https://www.bbc.co.uk/news/uk-wales-54598136.
4. BBC. Coronavirus: NI to face new lockdown measures from next Friday. 2020, November 19; Available from: https://www.bbc.co.uk/news/uk-northern-ireland-55004210.
5. BBC. Northern Ireland lockdown restrictions eased. 2021, May 24; Available from: https://www.bbc.co.uk/newsround/57225623.
6. CIMPSA. Updated guidance on sport, physical activity and leisure in Scotland – 2 November 2020. 2020, November 2; Available from: https://www.cimspa.co.uk/cimspa-news/news-home/updated-guidance-on-sport-physical-activity-and-leisure-in-scotland-%E2%80%93-2-november-2020.
7. GOV.UK. Further easing of COVID restrictions confirmed for 17 May. 2021, May 10; Available from: https://www.gov.uk/government/news/further-easing-of-covid-restrictions-confirmed-for-17-may.
8. Sport Scotland. Latest sport and physical activity guidance. 2021, July 7; Available from: https://sportscotland.org.uk/covid-19/latest-sport-and-physical-activity-guidance/.
9. The Executive Office. Executive tightens restrictions to curb Covid-19. 2020, October 14; Available from: https://www.executiveoffice-ni.gov.uk/news/executive-tightens-restrictions-curb-covid-19
10. The Health Foundation. COVID-19 policy tracker 2020. 2021; Available from: https://www.health.org.uk/news-and-comment/charts-and-infographics/covid-19-policy-tracker.
11. UK Active. Members Update – 7th December 2020. 2020, December 7; Available from: https://www.ukactive.com/wp-content/uploads/2020/12/Covid-restrictions-7th-December.pdf.
12. Welsh Government. Further coronavirus restriction relaxations brought forward. 2021, April 22; Available from: https://gov.wales/further-coronavirus-restriction-relaxations-brought-forward.
