# Supplementary material for: UK Adults’ Exercise Locations, Use of Digital Programs, and Associations with Physical Activity During the COVID-19 Pandemic: Longitudinal Analysis of Data From the Health Behaviours During the COVID-19 Pandemic Study
Source: JMIR Form Res. 2022 Jun 21;6(6):e35021. doi: 10.2196/35021 (PMC9217149; doi:10.2196/35021)
Supplement: Multimedia Appendix 2 [file formative_v6i6e35021_app2.docx]

## Multimedia Appendix 2 – Changes to the protocol

### Outcome measures

The protocol only specified MVPA and MSA guideline adherence as separate outcomes. After uploading the protocol it was decided that combined PA guideline adherence was important to include to aid interpretation and to reflect the WHO PA guidelines.

### Measures (predictors)

The protocol specified age as a continuous variable. However, assumption checks indicated a non-linear relationship of age with the log of the outcomes. Hence, age was categorized (<35, 35-64, >64 years of age) for all analyses.

### Analysis

The following changes to the analysis plan were made after the protocol had been uploaded: First, due to GLMM convergence issues in SPSS, these models were instead run in R using package *lme4* with use of the bobyqa optimizer and increased iterations [1]. Second, due to data sparseness in the within-person measurements, the GLMM models only included random intercepts and an unstructured variance-covariance matrix. Repeated measures were grand-mean centered instead of partitioning the variance into between- and within-person components using person-mean centering. Third, although the protocol specified models with the categorization exercising outside the home environment vs only inside as sensitivity analyses, the team decided that the analysis belonged in the main results section.

Reference

1. BBC. Coronavirus: Quarantine changes come into effect in NI. 2020, July 10; Available from: https://www.bbc.com/news/uk-northern-ireland-53354520.
