# Supplementary material for: UK Adults’ Exercise Locations, Use of Digital Programs, and Associations with Physical Activity During the COVID-19 Pandemic: Longitudinal Analysis of Data From the Health Behaviours During the COVID-19 Pandemic Study
Source: JMIR Form Res. 2022 Jun 21;6(6):e35021. doi: 10.2196/35021 (PMC9217149; doi:10.2196/35021)
Supplement: Multimedia Appendix 3 [file formative_v6i6e35021_app3.docx]

## Multimedia Appendix 3 – Details of measures

### Outcome measures

#### Exercise location at FU1, FU2, FU3 (outcome in RQ1 and RQ2)

Participants who reported engaging in any level of MVPA or MSA at the respective wave were asked: ‘Where have you been exercising in the past month?’ (1. Inside my house/garden, 2. Outside my house/garden, 3. Both inside and outside my house/garden). Categorized into any activity inside house/garden (1, 3) vs only outside (2) for main analysis, and any outside activity (2, 3) vs only inside (1).

#### Types of exercise at FU1, FU2, FU3 (outcome in RQ1)

Outside the home environment – asked of participants who responded ‘2’ or ‘3’on the question about exercise location: ‘What exercises are you usually doing outside your house? (select all that apply)’. The available answers were: ‘gentle walking’, ‘brisk walking’, ‘alternative walking-running’, ‘running’, ‘cycling, team sports’, ‘racket sports’, ‘swimming’, ‘weightlifting/crossfit’, ‘online/app-based fitness classes/program’, ‘other’. The following binary variables were created (i) gentle walking vs not, (ii) any brisk walking/alternate walking-running/running/cycling/swimming vs not, (iii) any team/racket sports vs not, (iv) weightlifting vs not, (v) online/app-based fitness classes/program vs not, (vi) other vs not.

Inside the home environment – asked of participants who responded ‘1’ or ‘3’ on the question about exercise location: ‘What exercises are you usually doing inside your house/garden?’ (Select all that apply)’. Possible answers were: ‘exercise DVD’, ‘online/app-based fitness classes/program’, ‘using indoor exercise equipment that I already had’, ‘using indoor exercise equipment that I bought/borrowed during COVID-19’, ‘doing bodyweight exercises without using an online class or app (i.e. your own workout)’, ‘other’, which were used as binary variables (yes/no).

#### Use of digital PA programs at FU1, FU2, FU3 (outcome in RQ2):

Affirmative answer to ‘Online/app-based fitness classes/program’ (either as part of indoor or outdoor exercising) vs. no.

#### MVPA at FU1, FU2, FU3 (outcome in RQ3):

Computed by multiplying number of sessions per week and average minutes as assessed in the following questions [35, 36]: ‘In the past month, on average, HOW MANY TIMES PER WEEK have you done 15 MINUTES or more of moderate or vigorous AEROBIC PHYSICAL ACTIVITY? (e.g. brisk walk, jogging, dancing, cycling for recreation or commute, swimming)? Do NOT include strength training.’ (with answers capped at 14+) and ‘In the past month, HOW LONG (in MINUTES) was your average session of moderate or vigorous AEROBIC PHYSICAL ACTIVITY? Do NOT include strength training.’ Categorized into 2 levels: meeting WHO MVPA recommendations (≥ 150 min/week) vs not (< 150 min).

#### MSA at FU1, FU2, FU3 (outcome in RQ3):

Assessed with the following question [35, 36]: ‘In the past month, on average, on HOW MANY DAYS PER WEEK have you done STRENGTH TRAINING?

Examples: Pilates, push-ups, squats, yoga, and exercises involving free weights (e.g. dumb bells or alternatives such as water bottles/cans), weight machines or elastic resistance band.’ Answers were capped at 4+.

Categorized into 2 levels: meeting WHO MSA recommendations (≥ 2 sessions/week) vs not (<2 sessions/week).

#### Combined WHO PA guideline adherence (outcome in RQ3):

Categorized into 2 levels: meeting both WHO MVPA and MSA recommendations vs not.

### Measures (predictors)

#### Time invariant predictors:

##### Demographics assessed at baseline

- Female gender vs other
- Age (<35, 35-64, >64)
- White ethnicity vs other
- At least 16 years+ education (3,4,5,6) vs other (0, No formal qualification | 1, GCSE/School certificate/O-level/CSE | 2, Vocational qualifications (e.g., NVQ1+2) | 3, A-level/Higher school certificate or equivalent (e.g., NVQ3) | 4, Bachelor degree or equivalent (e.g., NVQ4) | 5, Masters/PhD/PGCE or equivalent | 6, Other)
- Health condition limiting PA, no (2) vs other (1, 3); measured at baseline (Do you now suffer from any condition that limits you from engaging in physical activity, including walking or doing housework? 1, Yes | 2, No | 3, Prefer not to say)
- Living in England vs other UK countries
- Space to exercise comfortably inside one’s home or garden at FU2, FU3; Assessed with the question ‘What exercising set-up do you have at home?’ and affirmative answer to ‘space to exercise comfortably indoors (e.g. if the weather is poor)’. Dichotomized into no space on at least one time point (=reference) vs. all others.

#### Time variant predictors:

- Employed (1+2) vs not; measured at FU1, FU2, FU3 (1, Employed (full or part-time) | 2, Self-employed (full or part-time) | 3, Student | 4, Furloughed during COVID-19 | 5, Laid off during COVID-19 | 6, Unemployed since before COVID-19 | 7, Retired | 8, Homemaker, full-time parent or carer | 9, Unable to work due to disability | 10, Other)
- Total isolation vs. all others; measured at FU1, FU2, FU3; 1 vs. all others (Which type of COVID-19 induced isolation are you experiencing? 1,Total isolation/quarantine (not leaving the house for ANY reasons, not even to buy groceries or medications or to exercise) | 2, Some isolation (not leaving the house EXCEPT to buy essential items, such as groceries or medication or to exercise) | 3, General isolation but still go out to work (still go out to work and to buy essential items, such as groceries or medications or to exercise) | 4, No isolation (I am free to leave the house whenever I like, including participating in social gatherings or group sports, going to a bar or restaurant, travelling for leisure)
- BMI, continuous, measured at baseline, at FU2 and FU3 (prefer not to say | don’t know = missing); FU1 values were computed as the weighted mean from baseline and FU2 at 2:1 ratio to account for the difference in time intervals, or as the weighted mean from baseline and FU3 (5:1 ratio) where FU2 data were missing.
- High perceived risk of COVID-19 to one’s health (COVID-19 risk); measured at FU1, FU2, FU3; 1,2 vs. all other (What risk does COVID-19 pose to your health now? 1, Major risk | 2, Significant risk | 3, Moderate risk | 4, Minor risk | 5, No risk at all | 77, Don’t know)
- Smoking (current vs not), measured at FU1, FU2 and FU3
- Alcohol consumption per week (≥14 units vs all others [16]), measured at FU1, FU2 and FU3 (Assessed by multiplication of weekly drinking frequency and average number of units assessed with the questions: ‘How often did you have a drink containing alcohol in the past month?’ and ‘In the past month, how many units of alcohol did you drink on a typical day when you were drinking?’)

#### Time variable:

- Time in months; To account for the unequal time intervals between measurement points, time was coded as months (1, 3 and 6 months) and centered at zero (0, 2 and 5).

#### Time variant predictors in RQ3 only:

- Exercise location, inside home environment (vs outside only) and outside (vs inside only); measured at FU1, FU2, FU3
  - Time x location interaction
- Use of digital PA programs at FU1, FU2, FU3: yes vs. no
