# Supplementary material for: UK Adults’ Exercise Locations, Use of Digital Programs, and Associations with Physical Activity During the COVID-19 Pandemic: Longitudinal Analysis of Data From the Health Behaviours During the COVID-19 Pandemic Study
Source: JMIR Form Res. 2022 Jun 21;6(6):e35021. doi: 10.2196/35021 (PMC9217149; doi:10.2196/35021)
Supplement: Multimedia Appendix 4 [file formative_v6i6e35021_app4.docx]

## Multimedia Appendix 4 – Unweighted baseline characteristics of the analytic sample and participants lost to follow-up

|  | % (n) | |  |
| --- | --- | --- | --- |
|  | Analytic sample (n = 1938) | Sample lost to follow-up (n = 629) | *P* |
| Age |  |  |  |
| < 35 years old | 16.8 (325) | 37.0 (233) | <.001 |
| 35-64 years old | 66.5 (1289) | 54.5 (343) |  |
| > 64 years old | 16.7 (324) | 8.4 (53) |  |
| Female | 69.7 (1350) | 66.0 (415) | .083 |
| White ethnicity | 95.0 (1841) | 90.5 (569) | <.001 |
| 16+ years of education | 88.3 (1711) | 84.3 (530) | .008 |
| Employed | 55.5 (1076) | 58.6 (368) | <.001 |
| Condition limiting PA | 12.6 (242) | 14.1 (86) | .346 |
| Living in England | 86.3 (1672) | 83.8 (527) | .121 |
| Total isolation | 6.2 (119) | 7.2 (44) | .384 |
| High perceived risk from COVID-19 | 23.8 (457) | 18.8 (115) | .010 |
| Smoker | 13.4 (259) | 29.6 (186) | <.001 |
| High alcohol consumption | 18.6 (341) | 20.3 (111) | .355 |
| Meeting WHO PA recommendations at baseline |  |  |  |
| MVPA | 46.5 (868) | 41.7 (235) | .048 |
| MSA | 36.2 (676) | 36.6 (207) | .839 |
| Both | 20.9 (390) | 22.3 (126) | .457 |
| BMI; M (SD) | 26.1 (4.9) | 25.5 (5.2) | .020 |
