# Supplementary material for: UK Adults’ Exercise Locations, Use of Digital Programs, and Associations with Physical Activity During the COVID-19 Pandemic: Longitudinal Analysis of Data From the Health Behaviours During the COVID-19 Pandemic Study
Source: JMIR Form Res. 2022 Jun 21;6(6):e35021. doi: 10.2196/35021 (PMC9217149; doi:10.2196/35021)
Supplement: Multimedia Appendix 5 [file formative_v6i6e35021_app5.docx]

## Multimedia Appendix 5 – Unweighted descriptive statistics: exercise locations, use of digital programs, and meeting of WHO PA recommendations at FU1, FU2 and FU3.

|  | % (n)^a^ |  |  |
| --- | --- | --- | --- |
|  | FU1 (n = 1938) | FU2 (n = 1768) | FU3 (n = 1725) |
| Exercise location |  |  |  |
| Inside home environment | 15.4 (296) | 13.4 (232) | 13.3 (224) |
| Outside home environment | 31.4 (603) | 40.0 (693) | 36.4 (612) |
| Both inside and outside | 47.2 (908) | 39.3 (682) | 39.3 (661) |
| Use of digital PA programs | 30.3 (583) | 23.6 (409) | 20.2 (399) |
| Meeting WHO recommendations |  |  |  |
| MVPA | 44.7 (861) | 42.4 (736) | 41.3 (695) |
| MSA | 35.1 (675) | 33.0 (573) | 31.0 (522) |
| Both | 18.6 (359) | 16.2 (282) | 15.2 (255) |

^a^Percentages are valid percentages (i.e., excluding missingness). Some participants who were active in at least one wave but dropped to inactivity in each respective wave are included in the total, hence percentages to not add up to 100%.
