# Supplementary material for: UK Adults’ Exercise Locations, Use of Digital Programs, and Associations with Physical Activity During the COVID-19 Pandemic: Longitudinal Analysis of Data From the Health Behaviours During the COVID-19 Pandemic Study
Source: JMIR Form Res. 2022 Jun 21;6(6):e35021. doi: 10.2196/35021 (PMC9217149; doi:10.2196/35021)
Supplement: Multimedia Appendix 6 [file formative_v6i6e35021_app6.docx]

## Multimedia Appendix 6 – Unadjusted estimates: predictors of exercising inside (vs only outside), outside (vs only inside) the home environment, and of using digital PA programs (vs not) at FU1, FU2 and FU3

|  | Exercising  inside | Exercising  outside | Digital PA program use |
| --- | --- | --- | --- |
|  | OR  (95% CI) | OR  (95% CI) | OR  (95% CI) |
| Time^a^ (ref: FU1) |  |  |  |
| FU2 | 0.51 (0.42-0.62)*** | 1.50 (1.08-2.09) | 0.36 (0.27-0.48)*** |
| FU3 | 0.57 (0.47-0.70)*** | 1.17 (0.84-1.63) | 0.40 (0.30-0.53)*** |
| Age (ref: < 35 years) |  |  |  |
| 35-64 years | 0.62 (0.43-0.88)** | 1.30 (0.63-2.66) | 0.10 (0.05-0.20)*** |
| > 64 years | 0.74 (0.48-1.14) | 1.72 (0.70-4.23) | 0.03 (0.01-0.07)*** |
| Female gender (ref: all other) | 1.90 (1.45-2.49)*** | 1.01 (0.58-1.78) | 12.62 (8.11-19.65)*** |
| White ethnicity (ref: non-white) | 0.38 (0.21-0.70)** | 2.56 (0.77-8.54) | 0.16 (0.05-0.52)** |
| High education (ref: <16 years) | 1.14 (0.77-1.68) | 2.58 (1.16-5.73) | 5.96 (3.19-11.13)*** |
| Condition limiting PA (ref: none) | 1.68 (1.14-2.49)** | 0.24 (0.11-0.55)*** | 0.40 (0.21-0.75)** |
| England (ref: all other UK countries) | 1.37 (0.96-1.97) | 0.72 (0.33-1.57) | 1.85 (0.98-3.51) |
| Indoor space (ref: none) | 6.43 (4.89-8.47)*** | 0.68 (0.40-1.16) | 28.15 (16.81-47.14)*** |
| Employed (ref: not employed) | 0.91 (0.72-1.14) | 1.46 (0.94-2.28) | 1.67 (1.16-2.40)** |
| BMI | 0.95 (0.92-0.97)*** | 0.97 (0.93-1.03) | 0.85 (0.82-0.89)*** |
| High perceived risk of COVID-19 (ref: low) | 1.22 (0.94-1.58) | 0.47 (0.30-0.74)** | 0.63 (0.44-0.92)* |
| Total isolation (ref: not) | 6.39 (2.81-14.53)*** | 0.00 (0.00-0.02)*** | 1.81 (0.80-4.11) |

Significance after BH correction denotated by **P*<.05, ***P*<.01, ****P*<.001; ^a^BH-corrected significance level α=.03; ^b^ BH-corrected significance level α=.01; ^c^ BH-corrected significance level α=.04
