# Supplementary material for: UK Adults’ Exercise Locations, Use of Digital Programs, and Associations with Physical Activity During the COVID-19 Pandemic: Longitudinal Analysis of Data From the Health Behaviours During the COVID-19 Pandemic Study
Source: JMIR Form Res. 2022 Jun 21;6(6):e35021. doi: 10.2196/35021 (PMC9217149; doi:10.2196/35021)
Supplement: Multimedia Appendix 7 [file formative_v6i6e35021_app7.docx]

## Multimedia Appendix 7 – Complete case analysis: predictors of exercising inside (vs only outside), outside (vs only inside) the home environment, and of using digital PA programs (vs not) at FU1, FU2 and FU3

|  | Exercising  inside | Exercising  outside | Digital PA program use |
| --- | --- | --- | --- |
|  | OR  (95% CI) | OR  (95% CI) | OR  (95% CI) |
| Time^a^ (ref: FU1) |  |  |  |
| FU2 | 0.47 (0.37-0.59)*** | 1.37 (0.95-1.96) | 0.49 (0.36-0.65)*** |
| FU3 | 0.55 (0.43-0.70)*** | 0.99 (0.70-1.41) | 0.51 (0.38-0.68)*** |
| Age (ref: < 35 years) |  |  |  |
| 35-64 years | 0.63 (0.38-1.02) | 2.08 (0.90-4.78) | 0.22 (0.11-0.44)*** |
| > 64 years | 0.75 (0.42-1.35) | 4.90 (1.72-13.99)** | 0.09 (0.04-0.22)*** |
| Female gender (ref: all other) | 1.65 (1.17-2.32)** | 1.01 (0.55-1.88) | 10.17 (5.79-17.86)*** |
| White ethnicity (ref: non-white) | 0.33 (0.14-0.77)* | 3.33 (0.82-13.57) | 0.51 (0.17-1.52) |
| High education (ref: <16 years) | 0.62 (0.35-1.10) | 3.56 (1.31-9.68)* | 1.95 (0.82-4.68) |
| Condition limiting PA (ref: none) | 1.75 (1.02-2.98)* | 0.35 (0.14-0.89)* | 0.48 (0.21-1.10) |
| England (ref: all other UK countries) | 1.35 (0.86-2.11) | 0.58 (0.25-1.31) | 1.02 (0.51-2.01) |
| Indoor space (ref: none) | 6.46 (4.50-9.27)*** | 0.51 (0.29-0.91)* | 11.22 (6.58-19.14)*** |
| Employed (ref: not employed) | 1.12 (0.82-1.53) | 1.20 (0.72-2.02) | 1.32 (0.85-2.05) |
| BMI | 0.97 (0.94-1.00) | 0.97 (0.91-1.03) | 0.93 (0.88-0.98)** |
| High perceived risk of COVID-19 (ref: low) | 1.21 (0.87-1.67) | 0.56 (0.34-0.94)* | 1.01 (0.64-1.57) |
| Total isolation (ref: not) | 3.66 (1.24-10.75)* | 0.02 (0.00-0.07)*** | 0.88 (0.31-2.48) |

**P* <.05; ***P* <.01; ****P* <.001; ^a^N= 3225 observations, n= 1075 individuals
