# Supplementary material for: UK Adults’ Exercise Locations, Use of Digital Programs, and Associations with Physical Activity During the COVID-19 Pandemic: Longitudinal Analysis of Data From the Health Behaviours During the COVID-19 Pandemic Study
Source: JMIR Form Res. 2022 Jun 21;6(6):e35021. doi: 10.2196/35021 (PMC9217149; doi:10.2196/35021)
Supplement: Multimedia Appendix 8 [file formative_v6i6e35021_app8.docx]

## Multimedia Appendix 8 – Predictors of meeting MVPA, MSA and full recommendations (vs not) at FU1, FU2 and FU3, GLMM model estimates with key predictors for unadjusted models, adjusted using exercising inside (vs outside the home environment only, Model 1) and using exercising outside (vs inside the home environment only, Model 2)

|  | MVPA guideline adherence^a^ | | |
| --- | --- | --- | --- |
|  | Unadjusted | Model 1 | Model 2 |
|  | OR (95% CI) | OR (95% CI) | OR (95% CI) |
| Time | 0.84 (0.70-1.01) | 0.81 (0.67-0.97) | 0.81 (0.66-0.99) |
| Exercising inside (ref: outside only) | 0.56 (0.46-0.69)*** | 0.55 (0.41-0.73)*** | - |
| Exercising outside (ref: inside only) | 5.49 (4.02-7.50)*** | - | 4.74 (3.15-7.14)*** |
| Use of digital PA programs (ref: not)^d^ | 0.80 (0.64-1.01) | 1.05 (0.82-1.36) | 0.91 (0.71-1.15) |
| Time x location interaction | - | 0.99 (0.92-1.08) | 1.07 (0.94-1.22) |
|  | MSA guideline adherence^b^ | | |
|  | Unadjusted | Model 1 | Model 2 |
|  | OR (95% CI) | OR (95% CI) | OR (95% CI) |
| Time | 0.73 (0.58-0.91)** | 1.03 (0.81-1.30) | 0.86 (0.68-1.08) |
| Exercising inside (ref: outside only) | 13.71 (10.43-18.01)*** | 10.24 (6.94-15.13)*** | - |
| Exercising outside (ref: inside only) | 0.38 (0.27-0.53)*** | - | 0.42 (0.27-0.64)*** |
| Use of digital PA programs (ref: not) | 12.90 (9.53-17.50)*** | 5.09 (3.79-6.84)*** | 12.08 (8.92-16.35)*** |
| Time x location interaction | - | 0.88 (0.79-0.98)* | 1.11 (0.97-1.27) |
|  | Full guideline adherence^c^ | | |
|  | Unadjusted | Model 1 | Model 2 |
|  | OR (95% CI) | OR (95% CI) | OR (95% CI) |
| Time | 0.53 (0.39-0.72)*** | 0.73 (0.55-0.95)* | 0.65 (0.49-0.86)** |
| Exercising inside (ref: outside only) | 5.31 (3.85-7.32)*** | 4.09 (2.92-5.72)*** | - |
| Exercising outside (ref: inside only) | 2.13 (1.29-3.51)** | - | 2.11 (1.21-3.66)** |
| Use of digital PA programs (ref: not) | 4.59 (3.30-6.39)*** | 2.64 (1.90-3.68)*** | 4.85 (3.46-6.81)*** |
| Time x location interaction | - | 1.39 (1.21-1.59)*** | 1.11 (0.92-1.33) |

Significance after BH correction denotated by **P*<.05, ***P*<.01, ****P*<.001; ^a^BH-corrected significance level α=.02; ^b^ BH-corrected significance level α=.04; ^c^ BH-corrected significance level α=.05. ^d^BFs for associations of digital PA program use with MVPA guideline adherence were BF=0.93 (unadjusted), BF=0.20 (Model 1) and BF=0.23 (Model 2).
