# Supplementary material for: UK Adults’ Exercise Locations, Use of Digital Programs, and Associations with Physical Activity During the COVID-19 Pandemic: Longitudinal Analysis of Data From the Health Behaviours During the COVID-19 Pandemic Study
Source: JMIR Form Res. 2022 Jun 21;6(6):e35021. doi: 10.2196/35021 (PMC9217149; doi:10.2196/35021)
Supplement: Multimedia Appendix 10 [file formative_v6i6e35021_app10.docx]

## Multimedia Appendix 10 – Complete case analysis: predictors of meeting MVPA, MSA and full recommendations (vs not) at FU1, FU2 and FU3, GLMM model estimates with key predictors only and using exercising inside (vs outside the home environment only)

|  | MVPA^a^ | MSA^a^ | Full PA recommendations^a^ |
| --- | --- | --- | --- |
|  | OR  (95% CI) | OR  (95% CI) | OR  (95% CI) |
| Time | 0.82 (0.73-0.93)** | 0.96 (0.84-1.10) | 0.87 (0.75-1.01) |
| Exercising inside (ref: outside only) | 0.46 (0.31-0.67)*** | 9.05 (5.69-14.41)*** | 5.07 (3.05-8.44)*** |
| Use of digital PA programs (ref: not) | 0.89 (0.64-1.23) | 4.03 (2.84-5.72)*** | 1.98 (1.38-2.83)*** |
| Time x location interaction | 1.03 (0.93-1.15) | 0.88 (0.78-1.00) | 0.88 (0.76-1.01) |
| Age (ref: < 35 years) |  |  |  |
| 35-64 years | 2.97 (1.78-4.94)*** | 0.85 (0.49-1.46) | 2.09 (1.17-3.72)* |
| > 64 years | 2.45 (1.31-4.58)** | 0.53 (0.27-1.03) | 1.43 (0.71-2.90) |
| Female gender (ref: all other) | 0.60 (0.41-0.87)** | 0.45 (0.30-0.69)*** | 0.63 (0.41-0.96)* |
| White ethnicity (ref: non-white) | 1.78 (0.78-4.07) | 0.73 (0.30-1.76) | 0.94 (0.38-2.34) |
| High education (ref: <16 years) | 0.80 (0.44-1.45) | 0.85 (0.44-1.63) | 0.76 (0.39-1.49) |
| Condition limiting PA (ref: none) | 0.28 (0.16-0.50)*** | 1.02 (0.55-1.87) | 0.49 (0.25-0.96)* |
| England (ref: all other UK countries) | 0.62 (0.38-1.01) | 1.17 (0.69-1.98) | 0.65 (0.38-1.12) |
| Indoor space (ref: none) | 0.96 (0.67-1.37) | 2.59 (1.74-3.85)*** | 1.78 (1.19-2.66)** |
| Employed (ref: not employed) | 0.55 (0.40-0.76)*** | 0.71 (0.49-1.01) | 0.63 (0.43-0.91)* |
| BMI | 0.92 (0.89-0.96)*** | 0.88 (0.84-0.91) | 0.89 (0.85-0.93)*** |
| High perceived risk of COVID-19 (ref: low) | 0.85 (0.61-1.18) | 1.17 (0.81-1.70) | 0.99 (0.67-1.47) |
| Total isolation (ref: not) | 0.45 (0.18-1.08) | 1.56 (0.64-3.82) | 0.41 (0.13-1.25) |
| Smoker (ref: not) | 0.90 (0.52-1.55) | 0.40 (0.21-0.75)** | 0.48 (0.24-0.95)* |
| High alcohol consumption (ref: <14 units/week) | 0.79 (0.57-1.10) | 0.68 (0.47-0.98)* | 0.65 (0.44-0.97)* |

**P* <.05; ***P* <.01; ****P* <.001; ^a^N=3225 observations, n=1075 individuals. BFs for non-significant associations with digital PA program use were BF=0.14 (MVPA). ref=reference. OR=odds ratio. CI=confidence interval.
