# Supplementary material for: UK Adults’ Exercise Locations, Use of Digital Programs, and Associations with Physical Activity During the COVID-19 Pandemic: Longitudinal Analysis of Data From the Health Behaviours During the COVID-19 Pandemic Study
Source: JMIR Form Res. 2022 Jun 21;6(6):e35021. doi: 10.2196/35021 (PMC9217149; doi:10.2196/35021)
Supplement: Multimedia Appendix 11 [file formative_v6i6e35021_app11.docx]

## Multimedia appendix 11 – Posthoc analysis: associations of exercising inside the home environment with PA guideline adherence at FU1-FU3, results from fully adjusted binary logistic regression models

|  | FU1^a^ | FU2^b^ | FU3^c^ |
| --- | --- | --- | --- |
|  | OR  (95% CI) | OR  (95% CI) | OR  (95% CI) |
| MSA adherence |  |  |  |
| Exercising inside | 6.67 (4.75-9.36)*** | 4.48 (3.33-6.02)*** | 4.18 (3.04-5.75)*** |
| Full guideline adherence |  |  |  |
| Exercising inside | 3.71 (2.48-5.56)*** | 2.85 (1.97-4.13)*** | 2.41 (1.63-3.58)*** |

**P* <.05; ***P* <.01; ****P* <.001; ^a^N=1612, ^b^N=1461, ^c^N=1366.
