# Supplementary material for: UK Adults’ Exercise Locations, Use of Digital Programs, and Associations with Physical Activity During the COVID-19 Pandemic: Longitudinal Analysis of Data From the Health Behaviours During the COVID-19 Pandemic Study
Source: JMIR Form Res. 2022 Jun 21;6(6):e35021. doi: 10.2196/35021 (PMC9217149; doi:10.2196/35021)
Supplement: Multimedia Appendix 12 [file formative_v6i6e35021_app12.docx]

## Multimedia Appendix 12 – Predictors of meeting MVPA, MSA, and full recommendations (vs not) at FU1, FU2, and FU3. Unadjusted and fully adjusted GLMM estimates with key predictor exercising outside (vs inside the home environment only).

|  | MVPA^a^ | MSA^a^ | Full PA recommendations^a^ |
| --- | --- | --- | --- |
|  | OR  (95% CI) | OR  (95% CI) | OR  (95% CI) |
| Time | 0.82  (0.67-1.01) | 0.84  (0.67-1.06) | 0.68  (0.52-0.89)** |
| Exercising outside (ref: inside only) | 4.36  (2.87-6.63)*** | 0.42  (0.27-0.65)*** | 1.89  (1.10-3.23)* |
| Use of digital PA programs (ref: not) | 0.95  (0.74-1.23) | 8.71  (6.39-11.86)*** | 3.95  (2.84-5.50)*** |
| Time x location interaction | 1.07  (0.94-1.23) | 1.10  (0.96-1.26) | 1.11  (0.93-1.32) |
| Age (ref: < 35 years) |  |  |  |
| 35-64 years | 3.45  (2.35-5.08)*** | 1.03  (0.65-1.63) | 2.40  (1.41-4.09)*** |
| > 64 years | 2.59  (1.60-4.18)*** | 0.69  (0.38-1.25) | 1.65  (0.84-3.22) |
| Female gender (ref: all other) | 0.58  (0.43-0.77)*** | 0.50  (0.35-0.72)*** | 0.56  (0.37-0.84)** |
| White ethnicity (ref: non-white) | 1.19  (0.66-2.17) | 0.66  (0.32-1.38) | 0.65  (0.28-1.48) |
| High education (ref: <16 years) | 0.88  (0.58-1.32) | 1.09  (0.65-1.83) | 0.81  (0.45-1.47) |
| Condition limiting PA (ref: none) | 0.39  (0.25-0.59)*** | 0.82  (0.49-1.37) | 0.48  (0.26-0.89)* |
| England (ref: all other UK countries) | 0.53  (0.36-0.77)*** | 1.14  (0.71-1.83) | 0.59  (0.35-0.99)* |
| Indoor space (ref: none) | 1.05  (0.79-1.38) | 4.06  (2.82-5.83)*** | 2.59  (1.75-3.84)*** |
| Employed (ref: not employed) | 0.66  (0.51-0.86)** | 0.71  (0.52-0.97)* | 0.66  (0.46-0.94)* |
| BMI | 0.91  (0.88-0.93)*** | 0.89  (0.86-0.93)*** | 0.89  (0.85-0.93)*** |
| High perceived risk of COVID-19 (ref: low) | 0.95  (0.73-1.25) | 1.13  (0.82-1.56) | 0.92  (0.63-1.35) |
| Total isolation (ref: not) | 0.71  (0.34-1.46) | 1.32  (0.61-2.85) | 0.58  (0.21-1.63) |
| Smoker (ref: not) | 0.66  (0.45-0.97)* | 0.33  (0.20-0.54)*** | 0.39  (0.21-0.70)** |
| High alcohol consumption (ref: <14 units/week) | 0.79  (0.61-1.03) | 0.72  (0.52-0.99)* | 0.68  (0.47-0.98)* |

**P* <.05; ***P* <.01; ****P* <.001; ^a^N=4439 observations, n=1769 individuals. BFs for non-significant associations with digital PA program use were BF=0.14 (MVPA). ref=reference. OR=odds ratio. CI=confidence interval.
